# Cesarean sections and social inequalities in 305 cities of Latin America

**DOI:** 10.1016/j.ssmph.2022.101239

**Published:** 2022-09-27

**Authors:** Mónica Serena Perner, Ana Ortigoza, Andrés Trotta, Goro Yamada, Ariela Braverman Bronstein, Amélia Augusta Friche, Marcio Alazraqui, Ana V. Diez Roux

**Affiliations:** a Institute of Collective Health, National University of Lanus, Argentina; b CONICET (National Scientific and Technical Research Council), Argentina; c Urban Health Collaborative, Dornsife School of Public Health, Drexel University, USA; d Observatory for Urban Health in Belo Horizonte, School of Medicine, Federal University of Minas Gerais, Brazil

## Abstract

**Background:**

Cesarean section (CS) is a surgical procedure that, when medically justified, can help reduce maternal and infant morbidity and mortality. Worldwide CS rates (CSR) have been increasing; Latin America has rates that are among the highest in the world.

**Aim:**

Describe the variability of CSR across cities in Brazil, Colombia, Guatemala, Mexico, and Peru and examine the relationship of individual-level, sub-city, and city-level socioeconomic status (SES) with CSR.

**Methods:**

We used individual level data from vital statistics over the period 2014–2016 (delivery method, mother's age and education), census data to characterize sub-city SES and city GDP per capita from other sources compiled by the SALURBAL project. We fitted multilevel negative binomial regression models to estimate associations of SES with CSR.

**Results:**

11,549,028 live births from 1,101 sub-city units in 305 cities of five countries were included. Overall, the CSR was 52%, with a wide range across sub-cities (13–91%). Of the total variability in sub-city CSRs, 67% was within countries. In fully adjusted model higher CSR was associated with higher maternal education [(PRR (CI95%) 0.81 (0.80–0.82) for lower educational level, 1.32 (1.31–1.33) for higher level (ref. medium category)], with higher maternal age [PRR (CI95%) 1.23 (1.22–1.24) for ages 20–34 years, and 1.48 (1.47–1.49) for ages ≥ 35 years (ref. ≤19 years], higher sub-city SES [(PRR (CI95%) 1.02 (1.01–1.03) per 1SD)], and higher city GDP per capita [(PRR (CI95%): 1.03 (1.00–1.07) for GDP between 10,500–18,000, and 1.09 (1.06–1.13) for GDP 18,000 or more (ref. <10,500)].

**Conclusion:**

We found large variability in CSR across cities highlighting the potential role of local policies on CSR levels. Variability was associated in part with maternal and area education and GDP. Further research is needed to understand the reasons for this pattern and any policy implications it may have.

## Highlights

• Overall cesarean section rate was 52%, ranging across large cities from 13% to 91%.
• Lower cesarean rate was found in mothers with lower education (39%) vs higher (61%).
• Better socioeconomic conditions were associated with higher caesarean section rate.
• Reduction of caesarean rates needs concerted action on healthcare working process.

## 1 Introduction

Cesarean section (CS) is a standardized surgical procedure that when medically justified, such as for placenta previa or uterine rupture, can prevent maternal and perinatal mortality and morbidity. There is no evidence of benefits of caesarean delivery for women or infants when there is no medical indication for the procedure (WHO, 2015) but there is evidence of an increased risk of maternal complications (such as hysterectomy, hemorrhage, infections, and even death), and neonatal complications (respiratory and immunologic problems), as well as an increase of late preterm births and low birthweight (Gregory et al., 2012; Silva et al., 2010; Souza et al., 2010). Many of the conditions derived from unnecessary CSs may have long-term implications for women's health, and their future sexual and reproductive lives as well as for the health of their children (UNICEF, 2016; WHO, 2015). Beyond the potential risks for women and newborns, the additional costs of medically unnecessary CSs significantly impact healthcare expenditures (Betrán & YeMollerZhangGülmezogluTorloni, 2016; Rebelo et al., 2010; WHO, 2015; World Health Organization, 2009). Compared to vaginal deliveries, caesarean deliveries require higher numbers of medical personnel, more advanced levels of training from staff, and longer hospital stays for women (UNICEF, 2016).

In 1985 the World Health Organization (WHO) suggested that an acceptable cesarean section rate (CSR) based on medically necessary procedures is likely between 5 and 10–15 (10% for general populations, 15% for high-risk populations) (Wagner, 2000; WHO, 1985). This value was based on the CSR of countries with the lowest maternal and perinatal mortality in the world (the northern European countries) at the time (WHO, 2015; Ye et al., 2014). This threshold has been regarded by the international community as the “optimal” CSR and was confirmed in 2015 by WHO (WHO, 2015). Despite the lack of concrete evidence on what an appropriate threshold is (Betran et al., 2015; Ye et al., 2014), it is generally accepted that high CS rates need urgent attention (Ye et al., 2014), in part because of sharp increases in cesarean delivery the past decades, especially in Latin America (Betrán et al., 2016; Ye et al., 2014).

Worldwide, CSR has increased from 6.7% in 1990, to 19.1% in 2014 (185% increase). In Latin America, rates increased from 22.8% to 42.2%, with Latin America becoming the region with the largest absolute increase (19.4 points), and with the highest CSR (42.2%) among all world regions in 2014 (Betrán et al., 2016). The rising rate of elective cesarean deliveries suggests that both health-care workers and women perceive them as a safe procedure (World Health Organization, 2009). The increase is also likely influenced by cultural beliefs as well as socioeconomic and legal factors (Betrán & YeMollerZhangGülmezogluTorloni, 2016; Villar et al., 2007; Lin & Xirasagar, 2004), and also related to the use of surgical procedures in healthcare (Leone et al., 2008; Rothman, 2012). Differences in healthcare access, healthcare system factors, and approaches to perinatal care between and within countries may also influence the choices women or professionals make (Rebelo et al., 2010; Torres et al., 2014).

There are also important inequalities in the use of this procedure not only between countries but also within countries, and unnecessary CS impose important costs on financially stretched health systems (Betrán & YeMollerZhangGülmezogluTorloni, 2016). The term “cesarean paradox” (Althabe & Belizán, 2006) has been used to refer to the phenomenon by which underuse of CS among poorer women coexists with unnecessary use in those with higher income, both extremes with potential harmful consequences for women, newborns, and healthcare systems. There are also inequities across countries: while CSR have been found to be very low in very poor countries (Ronsmans et al., 2006; Showalter & Griffin, 1999), studies in low and middle income countries, including Latin American countries, have shown very high rates among wealthier and more highly educated women (Boatin et al., 2018; Rodgers et al., 2022; Ronsmans et al., 2006, Rebelo et al., 2010; Rodgers et al., 2022; World Health Organization, 2009). Higher national GDP has also been found to be associated with higher CSR (Showalter & Griffin, 1999; Ye et al., 2014).

Latin American countries differ in health care access and in the organization of healthcare which has consequences for pregnancy and birth care. One of the most urbanized regions of the world, over 80% of the population of Latin America lives in cities (World Bank Group, 2022). Cities may differ in CSR and city level factors may be related to heterogeneities in CSR across cities. However, few studies have described levels of CSR in Latin American cities, heterogeneities across cities or the extent to which city and maternal factors are related to CSR. Characterizing levels of CSR in cities and the factors associated with CSR is important for raising awareness among policy makers and identifying appropriate interventions and policies to reduce the harmful consequences of CSR under and overuse.

This study examined the proportion of cesarean births across 305 cities in 5 countries in Latin America as well as the association of cesarean births with socioeconomic characteristics of women and the cities in which they live.

## 2 Materials and methods

### 2.1 Data collection

Data was compiled as part of the *Salud Urbana en América Latina* (SALURBAL) project, which compiled health, social and built-environment data on all cities of 100,000 persons or more in 11 countries. Cities were defined as agglomerations of administrative units (i.e., *municipios*, *comunas*, *departamentos*, *delegaciones*, *corregimientos* or *distritos*) that encompassed the built-up urban extent of the city. Sub-cities were defined as administrative units nested within cities (Quistberg et al., 2019). This study is based on 305 cities in five countries with available data on delivery method in live birth records: Brazil, Colombia, Guatemala, Mexico, and Peru. The 305 cities include 1,101 sub-city units (of the 305 cities 147 cities include only one sub-city unit). We included live births for the period 2014–2016 for Brazil, Colombia, Mexico, and Peru, and 2015–2017 for Guatemala (since data for 2014 was not available).

### 2.2 Outcome and individual level characteristics

#### 2.2.1 Individual level data

Individual-level data included the following information on each birth: *delivery method* (CS yes or no), *mothers’ education* a proxy of individual-level socioeconomic status (SES) (less than primary; at least primary/less than secondary; completed secondary or above), *mothers’ age* (less than 19 years, 20–34, 35 or more), *birth weight* (less than 1,500 g, 1,500 to 2,500, 2,500 to 4,000, and more than 4,000 g), and *birth order* (first, second, third, fourth or more).

#### 2.2.2 Sub-city and city exposures

We explored a set of sub-city and city level exposures that we hypothesized could be associated to CSR based on prior work. We used a score of population educational attainment to characterize the social environment at the sub-city level, this index was derived and tested empirically in prior SALURBAL analyses (Ortigoza et al., 2021). This score included aggregate indicators of individual level of education retrieved from national census in each country: (1) percentage of population age 25 or above that has completed high school level or above, and (2) percentage of population age 25 or above that completed university level or above. Higher score values signify better educational achievement in the population. The measure was derived using principal component analysis of a larger set of indicators (Ortigoza et al., 2021) and was created by summing the standardized Z-scores for each of the two variables using the entire distribution (mean and standard deviation) of the entire sample of countries from the SALURBAL study. We included the index of population educational attainment in order to identify the contextual effect of education, given that individual education for mothers was also available. The year for which census data was available varied across countries: 2010 for Brazil, 2005 for Colombia, 2002 for Guatemala, 2010 for Mexico, and 2007 for Peru.

City-level exposures included gross domestic product per capita (GDP per capita) and population size. GDP per capita in constant 2011 international USD for the year 2015 was derived from modelling data for larger administrative units attributed to cities from Penn World Tables (Gennaioli et al., 2013; Kummu et al., 2018). Population size was obtained from population projections for the year 2015, it was log transformed for regression analysis.

Crude birth rates were calculated for each sub-city as number of live births per 1,000 population. Population data were from population projections for year 2015.

### 2.3 Statistical analysis

We examined the distribution of CSR in the overall sample and by countries. We performed descriptive analysis of distributions of CSR and main exposures. To estimate the proportion of variability in sub-city CSR between cities and between countries we fit a linear multilevel model with sub-city CSR as the dependent variable and random intercepts for city and country.

Maternal education was examined as a categorical variable in descriptive analyses but as continuous in regression analyses as the relation with CSR was approximately linear. GDP showed a non-linear association with CSR and was therefore included in models in three categories created based on inflections in the association with CSR (Supplementary Fig. 1).

We fitted a series of models to assess the association of maternal education as well as sub-city and city exposures with CS rates using multilevel negative binomial regression with robust variance estimation. A negative binomial model was used rather than a Poisson model to account for overdispersion. In addition, this negative binomial regression allows us to directly estimate prevalence ratios rather than odds ratios, recommended due the high prevalence of the outcome. For this purpose, number of live births delivered by cesarean and total number of live births counts were calculated for each cross-classified 3 × 3 cells (Lisabeth et al., 2007; Subramanian et al., 2001) of maternal age (less than 19, 20–34, and 35 and over), and maternal education (less than primary; at least primary, less than completed secondary; completed secondary and above). These cells were nested within sub-cities and these within cities. Records with missing data on delivery method (9,810; 0.08%), and mother's education or age (149,057; 1.27%) were excluded. Model 1 only included individual level (age and education) variables. Model 2 added sub-city exposures, and model 3 added city exposures. In all models we included a random intercept for each sub-city, and we accounted for the impact of country level factors by including countries as fixed effects. We calculated prevalence-rate ratios (PRR) estimates and corresponding 95% confidence intervals. We did all analysis in STATA® version 15.1, and figures with R version 4.0.2.

Since in some countries it is common practice to perform c-sections on successive births after a cesarean birth, we carried out a sensitivity analysis restricted to only first births (n = 4,933,024 live births). Similarly, because many conditions related to prematurity and fetal growth retardation require delivery by CS, we also performed a sub-analysis restricted to births between 2,500 and 4,000 g (n = 10,090,729 live births), considered normal birthweight and highly likely to be term newborns, as information on gestational age was not available on all vital records.

The SALURBAL study protocol was approved by the Drexel University IRB (ID#1612005035).

## 3 Results

A total of 11,549,028 live births from 1,101 sub-city units from 305 cities in five countries were studied.

**Fig. 1:**
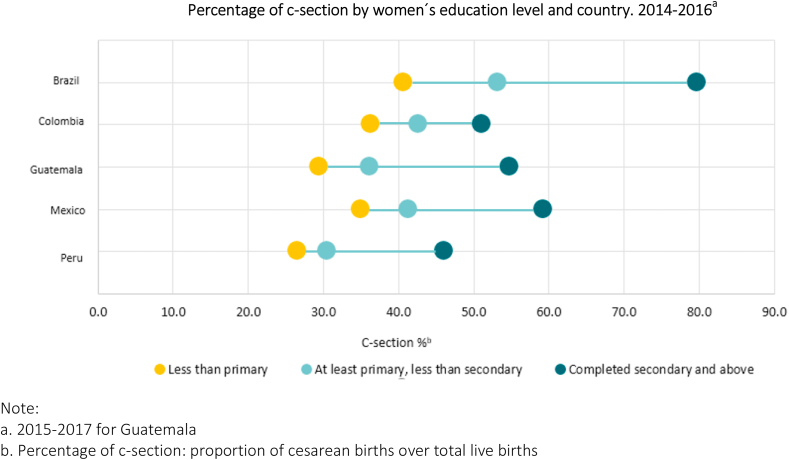
Distribution of c-section percentage by sub-city unit (n = 1,101) and country.

Table 1 shows the distribution of live births, c-sections, sub-city, and city characteristics by country. Together Brazil and Mexico account for 80.0% (9,234,893) of live births. The median number of live births per sub-city unit was 4,815 (10th −90th percentile: 684–22,685). Crude birth rates showed higher values for cities in Guatemala, followed by Peru, and Mexico. Overall, for the three-year period 2014–2016, 51.9% of live births were delivered by CS with an almost 20 percent point difference between sub-city medians across countries, ranging from 59.4% in Brazil to 40.1%, in Colombia. Overall, 86.3% of newborns weighed between 2,500 and 4,000 g, and 70.5% were born to mothers between 20 and 34 years. The proportion of live births to mothers 19 years or less ranged from 10.0 in Peru to 18.6 in Mexico (Supplementary Table 1). Overall, among mothers with lower level of education, the proportion of CS was 39.1% while among women with higher level of education it was 61.0%. These differences between low and high levels of maternal education were higher in Brazil, 40.7% vs 79.8%, respectively (Fig. 1).

**Table 1:** Distribution of live births, sub-city, and city level characteristics, in overall sample and by country. (2014–2016).

			Brazil	Colombia	Guatemala	Mexico	Peru			Total	
***Number of units of analysis***											
Number of cities			152	35	3	92	23			305	
Number of sub-city units			422	84	20	406	169			1,101	
Total number of live births			5,137,484	1,195,742	218,464	4,081,593	899,574			11,539,655	
***Sub-city-level characteristics*** [median (10th −90th percentile)]											
Live births^a^	5,574 (1,030–22,685)			5,166 (870–26,937)	6,508 (1,504–26,466)	3,978 (574–27,941)		3,010 (360–14,166)			4,815 (684–22,685)
Crude birth rate (per 1,000 population) b	43.7 (37.1–46.0)			41.5 (29.5–59.7)	62.6 (46.5–76.2)	52.0 (40.9–62.3)		53.0 (39.3–68.5)			48.0 (37.6–62.2)
Proportion of cesarean births c	59.4 (44.7–73.2)			40.1 (31.9–69.7)	45.4 (32.5–51.2)	48.9 (37.9–60.0)		40.6 (25.2–56.2)			50.3 (36.2–68.2)
Educational Attainment (z-score) d	−0.61 (−1.89/0.85)			−0.68 (−1.85/0.59)	−2.08 (−3.16/-0.50)	−1.19 (−2.48/0.94)		1.84 (0.19–5.09)			−0.62 (−2.14/1.82)
***City-level characteristics*** [median (10th −90th percentile)]											
Population^e^, thousands		244 (130–1,391)		337 (128–1,947)	288 (161–3,249)	381 (155–1,265)			303 (137–920)	300 (135–1,340)	
GDP per capita^f^		19,257 (1,715–24,921)		11,817 (6,372–16,410)	8,356 (5,707–17,056)	13,955 (8,617–21,002)			8,033 (5,588–15,355)	14,193 (5,673–24,921)	

Notes.

a Median live births and percentiles in sub-cities for three-year period 2014–2016 for Brazil, Colombia, Mexico, and Perú; 2015–2017 for Guatemala.

b Median crude birth rate in sub-cities: number of live births per 1,000 population (2015).

c Proportion of cesarean births in sub-cities for three-year period 2014–2016 for Brazil, Colombia, Mexico, and Perú; 2015–2017 for Guatemala.

d Educational attainment uses two census measured at population level: a. Proportion of the population aged 25 or older who completed secondary education or above. b. Proportion of the population aged 25 or older who completed university education or above. Measure was created with standardized z-scores of the two variables. Year of census: 2010 for Brazil, 2005 for Colombia, 2002 for Guatemala, 2010 for Mexico, and 2007 for Peru.

e Median and percentiles of total population in 2015 from cities over 100,000 inhabitants.

f Median and percentiles of GDP per capita from cities over 100,000 inhabitants in constant 2011 international USD for the year 2015.

**Fig. 2:**
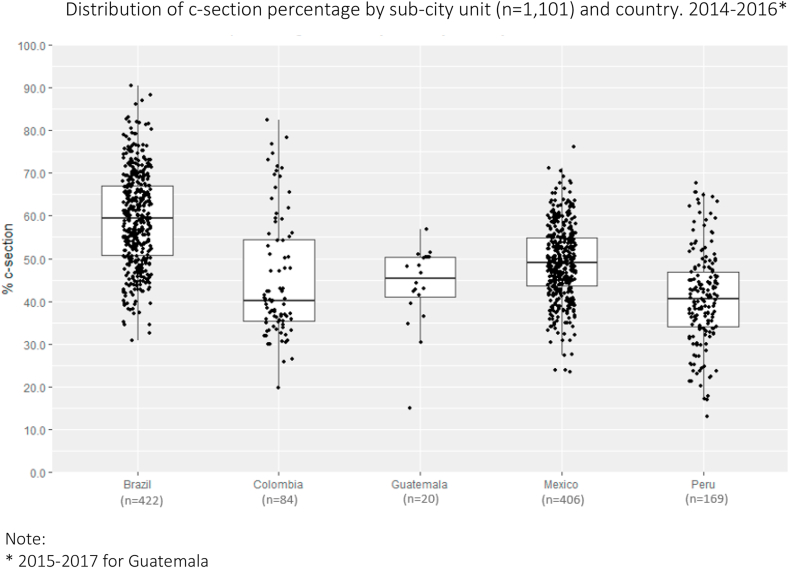
Distribution of c-section percentage by sub-city unit (n = 1,101) and country.

Fig. 2 shows the distribution of the proportion of c-section across sub-cities by country, showing large heterogeneity between and within countries: with a minimum value of 13.1% in a Peruvian sub-city, to a maximum of 90.5% in a Brazilian sub-city. Of the total variability in sub-city CSR, 25.0% was between sub-cities within cities, 42.5% between cities within a country, and 32.5% between countries.

Table 2 shows the proportion of CS according to maternal as well as sub-city and city level variables. In all countries the CSR was strongly patterned by maternal age and education with higher rates in more educated and in older mothers. In general CSR were also slightly higher at birthweight below 1,500g and above 4,000 g although the patterns were not consistent across all countries. In all countries CSR were higher among first births and progressively decreased as the number of previous births increased. CSR also increased as sub-city education increased in a linear fashion.

**Table 2:** Proportion of c-sections according to individual, sub-city, and city level variables. Overall and by country. 2014 a 2016^a^.

	Brazil	Colombia	Guatemala	Mexico	Peru	Total
	%	%	%	%	%	**%**
**Mothers’ education**						
Less than Primary	40.7	36.3	29.5	35.0	26.5	**39.1**
At least primary, less than sec.	53.2	42.6	36.2	41.4	30.6	**47.3**
Compl. Secondary and above	79.8	51.1	54.8	59.3	46.2	**61.0**
**Mothers' age at the time of birth**						
Less than or equal to 19 years	37.1	39.3	36.4	38.0	30.6	**37.4**
20–34 years	58.6	48.8	47.4	49.5	41.1	**52.7**
35 years or more	71.9	60.4	53.6	63.4	52.6	**66.0**
**Weight**						
<1,500 g	60.0	76.0	67.4	66.1	64.4	**63.6**
1,500–2,500 g	60.9	61.3	55.1	62.8	61.5	**61.3**
2,500–4,000 g	56.1	46.3	44.9	47.4	39.9	**50.5**
>4,000 g	69.4	64.4	53.6	57.3	57.1	**64.3**
**Birth order**						
First	59.2	47.1	49.8	51.4	45.7	**53.8**
Second	59.5	52.2	50.9	50.2	42.7	**53.8**
Third	54.2	48.5	43.9	47.4	40.4	**49.4**
Fourth or more	39.4	36.2	27.2	31.6	31.3	**35.2**
**Population Educational attainment**^b^						
Quartile 1	51.7	41.3	31.9	44.7	33.7	**45.6**
Quartile 2	54.2	47.8	44.3	48.3	40.5	**49.8**
Quartile 3	59.1	54.7	47.6	47.5	39.9	**51.2**
Quartile 4	58.2	47.3	51.0	50.2	51.2	**53.7**
**GDP per capita**^c^						
<10,500	55.7	55.4	46.3	46.5	38.5	**52.6**
10,500–18,000	54.4	45.9	46.6	49.0	43.0	**58.5**
>18,000	58.4	50.4	–	48.8	44.5	**53.9**

Notes.

a 2015–2017 for Guatemala.

b Population educational attainment uses two census measured at population level: a. Proportion of the population aged 25 or older who completed secondary education or above. b. Proportion of the population aged 25 or older who completed university education or above. Measure was created with standardized z-scores of the two variables.

c GDP (gross domestic product) per capita for each city per population, in constant 2011 international USD for the year 2015.

Table 3 shows the cesarean prevalence-rate ratios (PRR) associated with maternal, sub-city and city level factors. In model 1, mother's age and education were positively associated with CSR (PRR and 95%CI compared to at least primary/less than secondary: 0.81 (0.80–0.81) for less than primary, and 1.32 (1.31–1.33) for completed secondary or above and PRR and 95%CI compared to age ≤19 years 1.23 (95%CI 1.22–1.24) for ages 20–34 years, and 1.48 (95%CI 1.47–1.49) for ages ≥ 35 years).

**Table 3:** PRR of c-sections associated with individual, sub-city, and city level characteristics (5 Latin-American countries, 2014–2016).

	Model 1	Model 2		Model 3
	PRR (CI 95%)	PRR (CI 95%)		PRR (CI 95%)
**Mothers’ education**				
Less than Primary	0.81 (0.80–0.81)	0.81 (0.80–0.82)	0.81 (0.80–0.82)	
At least primary, less than secondary	1.00	1.00	1.00	
Completed Secondary and above	1.32 (1.31–1.33)	1.32 (1.31–1.33)	1.32 (1.31–1.33)	
**Mothers' age**				
Less than or equal to 19 years	1.00	1.00	1.00	
20–34 years	1.23 (1.22–1.24)	1.23 (1.22–1.24)	1.23 (1.22–1.24)	
35 years or more	1.48 (1.47–1.49)	1.48 (1.47–1.49)	1.48 (1.47–1.49)	
**Sub-city units' educational attainment**^a^				
Population Educational attainment (1SD)	–	1.03 (1.02–1.04)	1.02 (1.01–1.03)	
**Cities GDP per capita**^b^				
<10,500	–	–	1.00	
10,500–18,000	–	–	1.03 (1.00–1.07)	
>18,000	–	–	1.09 (1.06–1.13)	
**Cities population size**^c^				
Population size	–	–	0.98 (0.96–1.00)	

Notes: PRR: prevalence-rate ratio; CI 95%: confidence interval 95%; SD: standard deviation.

•Model 1 is adjusted for women's age and education.

•Model 2 is model 1 adjusted by sub-cities educational attainment.

•Model 3 is model 2 adjusted by cities GDP and population size.

All models included countries as fixed effects (coefficients not shown), and random intercept for each sub-city.

a Educational attainment. Sum of z-scores of a. Proportion of the population aged 25 or older who completed secondary education or above. b. Proportion of the population aged 25 or older who completed university education or above.

b GDP (gross domestic product) per capita for each city per population for 2015.

c City population size for 2015 is log transformed.

After accounting for maternal characteristics, higher sub-city educational attainment score was associated with higher cesarean prevalence (model 2), PRR per SD higher sub-city education: 1.03 (95%CI 1.02–1.04). Associations between maternal age and education did not substantially change when sub-city socioeconomic characteristics were added to the model.

In model 3, were we added city GDP and city population size: GDP was associated with CSRs: PRR of 1.03 (95%CI 1.00–1.07) in cities with GDP between 10,500–18,000, and 1.09 (95%CI 1.06–1.13) in cities with 18,000 or more (ref. <10,500). 1 SD greater population was associated lower CSR, although the association was not statistically significant, PRR 0.98 (95CI% 0.96–1.00).

Similar patterns of associations were found in sensitivity analysis using samples of only first births and births between 2,500 and 4,000 g (Supplementary Table 2).

## 4 Discussion

We examined levels of CSR across 305 cities in five countries of Latin America and associations of CSR with socioeconomic characteristic at the maternal, sub-city and city level. Overall, CS were the most common delivery method, with 52% of births delivered by section. However, there was large heterogeneity in CSR between sub-cities from a low of 13% to a high of 91%. Most of the variability in sub-city level CSR (67%) was within countries. Higher CSRs were associated with higher maternal age and education level, higher population educational attainment in sub-cities, and higher city GDP. Our findings highlight the importance of local contexts on the levels of CSs. Of note, in contrast to other maternal and perinatal outcomes for which better social environment is associated with lower frequency (GTR, 2017; UNICEF, 2016), we found that across the individual, sub-city, and city level higher SES and GDP were associated with more frequent c-sections.

One key finding of our analyses is the very high rate of CS observed across Latin American cities as well as the large heterogeneity observed across cities and sub-cities. In general, CSR in the Latin American cities, were substantially higher than the WHO's standard of 15%. This was true across maternal educational categories and across maternal age. Virtually all cities studied had CSR well over 15%. The 15% threshold applies to the general population of pregnant women, not to hospitals whose patients may have diverse characteristics that may vary from one facility to another (Dumont & Guilmoto, 2020; World Health Organization et al., 2009). Nevertheless, our analyses reveal extraordinarily high levels of CSR in the countries studied, clearly well above what is medically necessary, and with likely consequences for maternal health and birth outcomes.

The 15% standard may need further discussion and evaluation in a Latin American context, since these values were defined by the WHO in 1985 based on maternal and perinatal outcomes from the limited data available at that time, from Northern European countries in the 1980s (WHO, 2015). Nevertheless, the very high CSR that we observed has multiple implications for cost, safety, and medical practice. As CSR increases, the medical skills needed to perform vaginal deliveries can suffer (Dumont & Guilmoto, 2020), and a history of previous CS leads to CS in subsequent births leading to a vicious cycle that reinforces CS (Showalter & Griffin, 1999). As a surgical procedure, CS can lead to well documented complications in both women and children, as well as an increase of healthcare cost compared to vaginal deliveries (Souza et al., 2010; Ye et al., 2014). Despite the high frequency and the relative safety of cesarean delivery, it is still considered major surgery (Gregory et al., 2012). The widespread variations in CSR between sub-cities, ranging from 13 to 90% is also striking given that there are appropriate criteria with specific medical indications developed for this surgical procedure, usually standardized via clinical practice guidelines developed by scientific societies and health organizations (del Carmen et al., 2020).

We examined a range of socioeconomic exposures at various levels as predictors of CSR. Other studies in Latin America, and in other regions of the world, have examined ecological associations of CSR with country level variables such as GDP (Belizán et al., 1999; Boatin et al., 2018; Ye et al., 2014) and individual-level associations of maternal education with CSR (Boatin et al., 2018; Cesaroni et al., 2008; Rebelo et al., 2010; Rodgers et al., 2022; Ronsmans et al., 2006; WHO, 2015) finding positive associations between country GDP and CSR and higher CSRs among wealthier women and among women with higher educational level. None of these studies analyzed data at a city or sub-city level. One study from India included other sub-national levels to account for variance at each one but did not examine explanatory variables at these levels (Rodgers et al., 2022). We were able to investigate the role of socioeconomic factors at three levels simultaneously: maternal education, sub-city educational attainment and city GDP after adjustment for each other and found that they were each independently associated with higher levels of CSR. To our knowledge, this is the first study examining associations of socioeconomic characteristics with CSR rates in urban areas.

The strong patterning of CSR by maternal education may reflect a number of factors including the place where the birth occurs, the type of provider, health care system incentives, cultural norms and maternal “preferences” (potentially influenced by norms and the health care system itself). More research is needed to better understand the drivers of these large differences. The associations of sub-city and city level factors with CSR may also reflect health care system or sociocultural factors. An important finding is that the sub-city and city level factors that we examined were only weakly associated with CSR suggesting that other unmeasured city, sub-city and individual-level factors may contribute to the significant heterogeneity in CSR that we observed across sub-cities within cities and across cities.

The patterns we observed (high rates of CS, extreme in some sub-cities, large heterogeneities between sub-cities and positive associations of SES at various levels with CSR) likely reflect complex medical and social processes (Showalter & Griffin, 1999). Cesarean sections should be viewed not only as a medical procedure, but also as a social practice of women and health-care professionals that is influenced by the social world that structures and organizes the health care delivery process. The practice of CS is likely impacted by the work process in healthcare facilities, as well as by broader sociocultural factors that impact women and health care providers. Studies of overuse of CS are relatively recent (Betrán et al., 2018), but it has been suggested that non-clinical factors such as behavioral, cultural, contextual, healthcare system and health professional-level factors may be important drivers which should be examined (Betrán et al., 2018; Hopkins, 2000; Leone et al., 2008).

The data we have available does not allow us to identify the causal processes underlying the associations that we observed. However, based on other work several reflections are possible. The healthcare work process is likely to play an important role in decision-making in the context of health care. Birth has become an institutionalized practice, controlled by physicians, but internalized and demanded by patients (Menéndez, 2005) such that the “natural” way women give birth is heavily influenced by medical practice. Childbirth is one example of a condition which remains firmly under medical control, and physicians legitimately lay claim to all activities concerning the condition (Conrad, 1992). Providers and healthcare organizations have incentives to prefer technology that supplies constant monitoring and active intervention, often resulting in an overuse of technology and in the medicalization (Conrad, 1992) of pregnancy and birth (Rosemberg et al., 2020; UNICEF, 2016), driven by health commodification and by economic interests over health (Cernadas, 2019).

Decisions made by healthcare workers are also strongly affected by concerns of possible legal consequences. Obstetricians and gynecologists are among the medical professionals with highest litigation risk (Rudey et al., 2021), which makes medical decisions, such as indicating a CS, as a defensive method to avoid potential litigation. It has been reported that vaginal births are associated with a higher risk of lawsuits when complications do occur than CS (Wagner, 2000; Rudey et al., 2021; Shwayder, 2007; Kravitz et al., 1991). In addition, based on the belief that CS makes deliveries more predictable, controllable, and more easily monitored (Cartwright, 2016), a scheduled cesarean delivery is a potential time-management solution (Lin & Xirasagar, 2004; Rebelo et al., 2010). Research in Latin America has found higher CSR in private than in public hospitals (Hopkins, 2000; Murray, 2000). Thus, differences in the use of private and public facilities across sub-cities and cities may also partly explain the associations that we observed.

Birth medicalization, potential litigation, and institutional organization impact the health care work process, conditioning delivery modes. In addition, sociocultural factors can impact the choices women make. Sociocultural factors (including time-use, fear of pain, and social norms) (UNICEF, 2016) impact women's’ attitudes towards obstetric interventions as well their choices in the birth delivery process. CS have been promoted as a practice that increases women's power to choose. But this choice is not as free as it appears and can also have important consequences that are not always made visible.

Our study has several limitations. It is descriptive in nature and does not shed light on the underlying causal processes involved. Only a limited set of constructs at the maternal, sub-city and city level were explored and many important factors (beliefs, health care system access and organization among others) were not considered. However, our findings suggest future avenues for exploration including health care-system factors and sociocultural norms. Sub-city educational attainment was retrieved from censuses for different years that were not always aligned with the years for which live births were obtained. We therefore assumed that population educational attainment measures were relatively stable across studied time. Although undercounting of births in Latin America is around 6%, and this is higher in areas with lower SES, the coverage of vital statistics registration is better in urban settings compared to rural areas, and the countries we included tend to have lower proportions of undercounting (less than 6%) (UNICEF, 2016). Several countries represented in the SALURBAL study (11 in total) could not be included because either birth information or delivery method information was not available. We also lacked information on several important variables that may explain differences in CSR by education, sub-city, and city level factors such as the use or public or private facilities, health insurance and type of provider, among others.

This study also has several strengths. To our knowledge this is the first examination of CSR variability across a large sample of diverse cities from the Latin American region. We included a large sample of live births (more than 11 million), representing all cities of 100,000 residents or more across 5 countries in the region. The multilevel structure allowed us to assess how much sub-city and city context contribute to the CSR variability across countries, while accounting for individual and maternal characteristics. Further research including qualitative approaches is required to provide better insight into the drivers of the patterns we report and to identify policy implications.

## 5 Conclusion

Our results suggest the need to further examine how local policies, practices and cultures impact CSR and further examine what proportion of CS are medically necessary. Of note our findings of associations of higher socioeconomic status with higher CS are compatible with important inequities in access to CS reflecting under use in medically necessary cases coupled with overuse in the population as a whole. Factors that may be driving the patterns we observed include the overuse of technology and the medicalization of birth, practices first promoted through the healthcare system, but then internalized and demanded by patients. Rather than reflecting the free choices of women and professionals, decisions to perform a CS are influenced by the medicalization of births and by the commodification of health care. Therefore, it is critical to consider the process of healthcare delivery and the economic systems driving healthcare use. Strategies may include interventions that focus on healthcare workers and institutions and healthcare payment strategies in order to rationalize the use of CS so that they are used appropriate to true need in order to ensure equitable access to medically necessary CSs, and thus improve the health of women and newborns.

## Funding

Funding This work was supported by the 10.13039/100010269Wellcome Trust initiative ‘Our Planet, Our Health’ (grant 205177/Z/16/Z). The study funder had no role in study design, data collection, data analysis, data interpretation or writing of this study. The corresponding author had full access to all the data in the study and had final responsibility for the decision to submit for publication.

## Ethical statement

The SALURBAL study protocol was approved by the Drexel University IRB (ID#1612005035).

## Credit author statement

Perner Mónica Serena: Conceptualization, Methodology, Software, Formal analysis, Data Curation, Writing - Original Draft. Visualization.

Ortigoza Ana: Methodology, Writing - Review & Editing.

Trotta Andrés: Conceptualization, Methodology, Writing - Review & Editing.

Yamada Goro: Methodology, Software, Writing - Review & Editing.

Braverman Ariela. Writing - Review & Editing.

Friche Amélia Augusta. Writing - Review & Editing.

Alazraqui Marcio: Conceptualization, Methodology, Writing - Review & Editing. Project administration. Supervision.

Diez Roux Ana V. Methodology. Writing - Review & Editing. Funding acquisition. Project administration. Supervision.

## Declaration of competing interest

None.

## Data Availability

Data will be made available on request.
