# Supplementary material for: Cesarean sections and social inequalities in 305 cities of Latin America
Source: SSM Popul Health. Author manuscript; Available in PMC 2022 Oct 13. (PMC9529579; doi:10.1016/j.ssmph.2022.101239)

Cesarean sections and social inequalities in 305 cities of Latin America

Supplementary table 1: Live births in cities of 5 Latin American countries according to selected variables. 2014 – 2016[a].

	Brazil (n= 5,193,520)		Colombia (n=1,231,717)		Guatemala (n=225,024)		Mexico (n=4,147,652)		Peru (n=900,172)		**Total (n=11,698,085)**	
	%	(n)	%	(n)	%	(n)	%	(n)	%	(n)	%	(n)
Delivery method												
Spontaneous	42.7	(2,218,764)	49.8	(613,903)	53.6	(120,494)	49.8	(2.065,455)	57.3	(516,027)	**47.3**	**(5,534,643)**
C-section	57.2	(2,970,737)	48.6	(598,506)	46.5	(104,530)	48.7	(2.018,254)	42.3	(380,754)	**51.9**	**(6,072,781)**
Other instruments	--		1.6	(19,229)	--		1.4	(58,552)	0.3	(3,070)	**0.7**	**(80,851)**
Missing	0.1	(4,019)	0.0	(79)	--		0.1	(5,391)	0.1	(321)	**0.1**	**(9,810)**
Birth weight												
<1500 g	1.5	(76,979)	1.3	(15,499)	1.1	(2,454)	0.7	(28,434)	0.8	(7,238)	**1.1**	**(130,604)**
1500-2500	7.5	(386,766)	8.3	(102,516)	13.3	(29,974)	5.6	(230,841)	4.7	(41,845)	**6.8**	**(791,942)**
2500-4000	86.3	(4,480,508)	87.8	(1,081,343)	84.0	(188,958)	85.7	(3,555,426)	87.2	(784,494)	**86.3**	**(10,090,729)**
>4000	4.8	(248,746)	2.6	(31,735)	1.6	(3,500)	2.7	(110,209)	7.3	(65,793)	**3.9**	**(459,983)**
Out of Range	0.0	(261)	--		--		0.0	(4)		--	**0.0**	**(265)**
Missing	0.0	(260)	0.1	(624)	0.1	(138)	5.4	(222,738)	0.1	(802)	**1.9**	**(224,562)**
**Mothers'** education at the time of birth												
Less than Primary	16.7	(866,185)	1.8	(21,764)	10.7	(23,993)	3.9	(161,225)	3.9	(35,007)	**9.5**	**(1,108,174)**
At least primary, less than secondary	59.8	(3,103,617)	28.5	(350,662)	28.4	(63,933)	53.0	(2,199,146)	19.9	(179,134)	**50.4**	**(5,896,492)**
Completed Secondary and above	22.6	(1,171,541)	66.9	(823,367)	58.0	(130,585)	41.9	(1,736,130)	76.2	(685,761)	**38.9**	**(4,547,384)**
Missing	1.0	(52,177)	2.9	(35,924)	2.9	(6,513)	1.2	(51,151)	0.1	(270)	**1.3**	**(146,035)**
**Mothers'** age at the time of birth												
Less than or equal to 19 years	15.6	(809,195)	18.5	(228,038)	15.4	(34,567)	18.6	(772,831)	10.0	(90,215)	**16.5**	**(1,934,846)**
20-34 years	69.8	(3,625,000)	70.5	(868,620)	73.6	(165,549)	71.3	(2,959,065)	70.4	(633,967)	**70.5**	**(8,252,201)**
35 years or more	14.6	(759,250)	11.0	(134,950)	11.0	(24,844)	9.9	(412,675)	19.6	(175,979)	**12.9**	**(1,507,698)**
Missing	0.0	(75)	0.0	(109)	0.1	(64)	0.1	(3,081)	0.0	(11)	**0.1**	**(3,340)**
Delivery setting in a health care facility												
Yes	99.6	(5,172,174)	99.7	(1,228,015)	87.5	(196,857)	99.4	(4,123,378)	98.7	(888,383)	99.2	**(11.608,807)**
No	0.4	(21,208)	0.3	(3,664)	6.0	(13,497)	0.6	(24,103)	1.3	(11,789)	0.6	**(74,261)**
Missing	0.0	(138)	0.0	(38)	6.5	(14,670)	0.0	(171)		--	0.1	**(15,017)**

Notes: [a] Data for Guatemala 2015-2017

Supplementary Table 2: Estimated PRR of c-sections associated with individual, sub-city, and city level characteristics. Sensitivity analysis			
	All live births (n=11,698,085)	Live births between 2500-4000 grams (n=10,090,729)	Women´s first birth (n=10,090,729)
	PRR (95% CI)	PRR (95% CI)	PRR (95% CI)
**Mothers´ education**			
Less than Primary	0.81 (0.80-0.82)	0.80 (0.79-0.81)	0.90 (0.89-0.91)
At least primary, less than secondary	1.00	1.00	1.00
Compl. Secondary and above	1.32 (1.31-1.33)	1.35 (1.34-1.36)	1.19 (1.18-1.20)
**Mothers´ age**			
Less than or equal to 19 years	1.00	1.00	1.00
20-34 years	1.23 (1.22-1.24)	1.23 (1.22-1.24)	1.38 (1.37-1.40)
35 years or more	1.48 (1.47-1.49)	1.48 (1.47-1.50)	1.87 (1.84-1.89)
**Sub-cities units educational attainment [a]**			
Population educational attainment (1SD)	1.02 (1.01-1.03)	1.02 (1.01-1.03)	1.02 (1.01-1.02)
**Cities GDP per capita[b]**			
<10,500	1.00	1.00	1.00
10,500-18,000	1.03 (1.00-1.07)	1.03 (1.00-1.07)	1.02 (1.00-1.05)
>18,000	1.09 (1.06-1.13)	1.10 (1.06-1.14)	1.08 (1.05-1.11)
**Cities population size[c]**			
Population size	0.98 (0.96-1.00)	0.98 (0.96-1.00)	0.97 (0.95-0.98)

Notes:

- PRR = prevalence-rate ratios. 95% CI = 95% confidence interval
- All models were adjusted for women´s age and education with sub-cities educational attainment, cities GDP and population size as random effects.

a. Educational attainment. Sum of z-scores of a. Proportion of the population aged 25 or older who completed secondary education or above. b. Proportion of the population aged 25 or older who completed university education or above.

b. GDP (gross domestic product) per capita for each city per population for 2015.

c. City population size for 2015 is log transformed.

Supplementary Figure 1: Relation between cesarean section rates and socioeconomic variables.

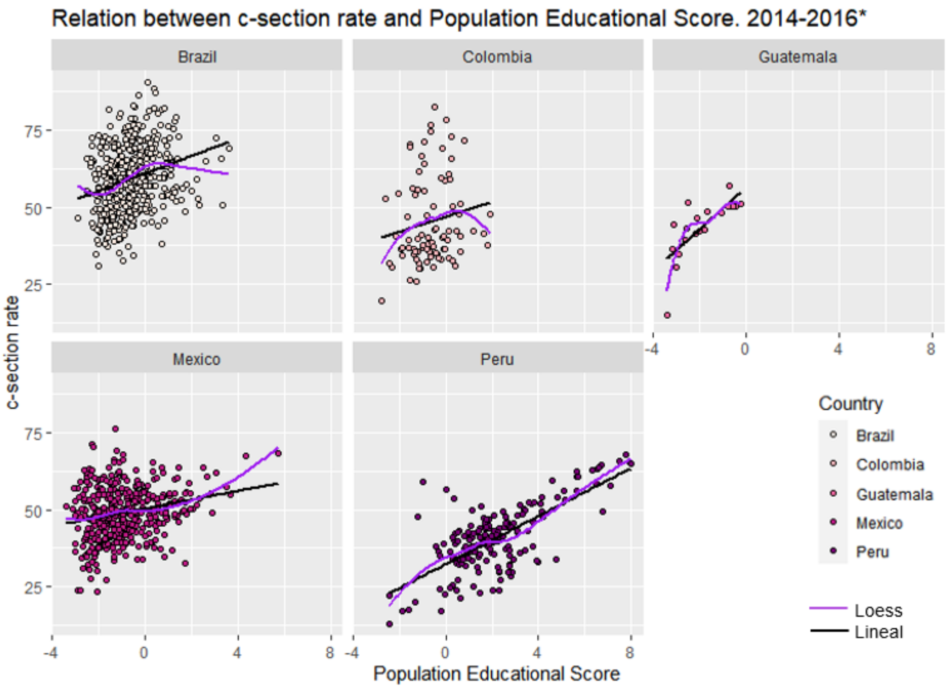

Not

* 2015-2017 for Guatemala

Population educational attainment uses two census measured at population level: a. Proportion of the population aged 25 or older who completed secondary education or above. b. Proportion of the population aged 25 or older who completed university education or above. Measure was created with standardized z-scores of the two variables.

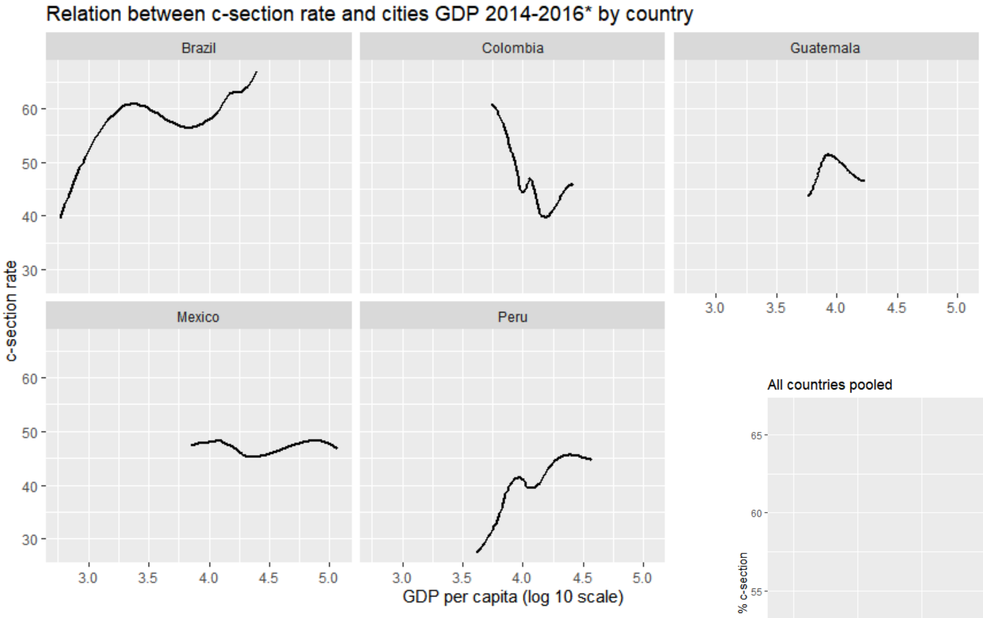

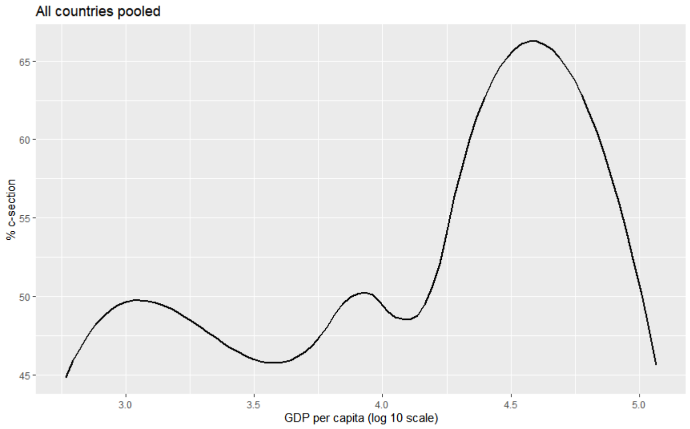

Supplement: Supplementary Data [file EMS155291-supplement-Supplementary_Data_.pdf]
