# Supplementary material for: Cesarean sections and social inequalities in 305 cities of Latin America
Source: SSM Popul Health. 2022 Sep 27;19:101239. doi: 10.1016/j.ssmph.2022.101239 (PMC9529579; doi:10.1016/j.ssmph.2022.101239)
Supplement: Supplementary file 1 [file mmc1.docx]

**Cesarean sections and social inequalities in 305 cities of Latin America**

|  | **Supplementary table 1: Live births in cities of 5 Latin American countries according to selected variables. 2014 – 2016**^**a**^. |  |  |  |  |  |  |  |  |  |  |  |  |
| --- | --- | --- | --- | --- | --- | --- | --- | --- | --- | --- | --- | --- | --- |
|  |  | Brazil |  | Colombia |  | Guatemala |  | Mexico (n=4,147,652) |  | Peru (n=900,172) |  | **Total** **(n=11,698,085)** |  |
|  |  | (n= 5,193,520) |  | (n=1,231,717) |  | (n=225,024) |  |  |  |  |  |  |  |
|  |  | % | (n) | % | (n) | % | (n) | % | (n) | % | (n) | % | **(n)** |
| **Delivery method** |  |  |  |  |  |  |  |  |  |  |  |  |  |
|  | Spontaneous | 42.7 | (2,218,764) | 49.8 | (613,903) | 53.6 | (120,494) | 49.8 | (2.065,455) | 57.3 | (516,027) | **47.3** | **(5,534,643)** |
|  | C-section | 57.2 | (2,970,737) | 48.6 | (598,506) | 46.5 | (104,530) | 48.7 | (2.018,254) | 42.3 | (380,754) | **51.9** | **(6,072,781)** |
|  | Other instruments | -- |  | 1.6 | (19,229) | -- |  | 1.4 | (58,552) | 0.3 | (3,070) | **0.7** | **(80,851)** |
|  | Missing | 0.1 | (4,019) | 0.0 | (79) | -- |  | 0.1 | (5,391) | 0.1 | (321) | **0.1** | **(9,810)** |
| **Birth weight** |  |  |  |  |  |  |  |  |  |  |  |  |  |
|  | <1500 g | 1.5 | (76,979) | 1.3 | (15,499) | 1.1 | (2,454) | 0.7 | (28,434) | 0.8 | (7,238) | **1.1** | **(130,604)** |
|  | 1500-2500 | 7.5 | (386,766) | 8.3 | (102,516) | 13.3 | (29,974) | 5.6 | (230,841) | 4.7 | (41,845) | **6.8** | **(791,942)** |
|  | 2500-4000 | 86.3 | (4,480,508) | 87.8 | (1,081,343) | 84.0 | (188,958) | 85.7 | (3,555,426) | 87.2 | (784,494) | **86.3** | **(10,090,729)** |
|  | >4000 | 4.8 | (248,746) | 2.6 | (31,735) | 1.6 | (3,500) | 2.7 | (110,209) | 7.3 | (65,793) | **3.9** | **(459,983)** |
|  | Out of Range | 0.0 | (261) | -- |  | -- |  | 0.0 | (4) |  | -- | **0.0** | **(265)** |
|  | Missing | 0.0 | (260) | 0.1 | (624) | 0.1 | (138) | 5.4 | (222,738) | 0.1 | (802) | **1.9** | **(224,562)** |
| **Mothers’ education at the time of birth** |  |  |  |  |  |  |  |  |  |  |  |  |  |
|  | Less than Primary | 16.7 | (866,185) | 1.8 | (21,764) | 10.7 | (23,993) | 3.9 | (161,225) | 3.9 | (35,007) | **9.5** | **(1,108,174)** |
|  | At least primary, less than secondary | 59.8 | (3,103,617) | 28.5 | (350,662) | 28.4 | (63,933) | 53.0 | (2,199,146) | 19.9 | (179,134) | **50.4** | **(5,896,492)** |
|  | Completed Secondary and above | 22.6 | (1,171,541) | 66.9 | (823,367) | 58.0 | (130,585) | 41.9 | (1,736,130) | 76.2 | (685,761) | **38.9** | **(4,547,384)** |
|  | Missing | 1.0 | (52,177) | 2.9 | (35,924) | 2.9 | (6,513) | 1.2 | (51,151) | 0.1 | (270) | **1.3** | **(146,035)** |
| **Mothers’ age at the time of birth** |  |  |  |  |  |  |  |  |  |  |  |  |  |
|  | Less than or equal to 19 years | 15.6 | (809,195) | 18.5 | (228,038) | 15.4 | (34,567) | 18.6 | (772,831) | 10.0 | (90,215) | **16.5** | **(1,934,846)** |
|  | 20-34 years | 69.8 | (3,625,000) | 70.5 | (868,620) | 73.6 | (165,549) | 71.3 | (2,959,065) | 70.4 | (633,967) | **70.5** | **(8,252,201)** |
|  | 35 years or more | 14.6 | (759,250) | 11.0 | (134,950) | 11.0 | (24,844) | 9.9 | (412,675) | 19.6 | (175,979) | **12.9** | **(1,507,698)** |
|  | Missing | 0.0 | (75) | 0.0 | (109) | 0.1 | (64) | 0.1 | (3,081) | 0.0 | (11) | **0.1** | **(3,340)** |
| **Delivery setting in a health care facility** |  |  |  |  |  |  |  |  |  |  |  |  |  |
|  | Yes | 99.6 | (5,172,174) | 99.7 | (1,228,015) | 87.5 | (196,857) | 99.4 | (4,123,378) | 98.7 | (888,383) | 99.2 | **(11.608,807)** |
|  | No | 0.4 | (21,208) | 0.3 | (3,664) | 6.0 | (13,497) | 0.6 | (24,103) | 1.3 | (11,789) | 0.6 | **(74,261)** |
|  | Missing | 0.0 | (138) | 0.0 | (38) | 6.5 | (14,670) | 0.0 | (171) |  | -- | 0.1 | **(15,017)** |
| Notes: ^a^ Data for Guatemala 2015-2017 |  |  |  |  |  |  |  |  |  |  |  |  |  |

| **Supplementary Table 2: Estimated PRR of c-sections associated with individual, sub-city, and city level characteristics. Sensitivity analysis** |  |  |  |
| --- | --- | --- | --- |
|  | All live births (n=11,698,085) | Live births between 2500-4000 grams (n=10,090,729) | Women´s first birth (n=10,090,729) |
|  | PRR (95% CI) | PRR (95% CI) | PRR (95% CI) |
| **Mothers´ education** |  |  |  |
| Less than Primary | 0.81 (0.80-0.82) | 0.80 (0.79-0.81) | 0.90 (0.89-0.91) |
| At least primary, less than secondary | 1.00 | 1.00 | 1.00 |
| Compl. Secondary and above | 1.32 (1.31-1.33) | 1.35 (1.34-1.36) | 1.19 (1.18-1.20) |
| **Mothers´ age** |  |  |  |
| Less than or equal to 19 years | 1.00 | 1.00 | 1.00 |
| 20-34 years | 1.23 (1.22-1.24) | 1.23 (1.22-1.24) | 1.38 (1.37-1.40) |
| 35 years or more | 1.48 (1.47-1.49) | 1.48 (1.47-1.50) | 1.87 (1.84-1.89) |
| **Sub-cities units educational attainment** ^**a**^ |  |  |  |
| Population educational attainment (1SD) | 1.02 (1.01-1.03) | 1.02 (1.01-1.03) | 1.02 (1.01-1.02) |
| **Cities GDP per capita**^**b**^ |  |  |  |
| <10,500 | 1.00 | 1.00 | 1.00 |
| 10,500-18,000 | 1.03 (1.00-1.07) | 1.03 (1.00-1.07) | 1.02 (1.00-1.05) |
| >18,000 | 1.09 (1.06-1.13) | 1.10 (1.06-1.14) | 1.08 (1.05-1.11) |
| **Cities population size**^c^ |  |  |  |
| Population size | 0.98 (0.96-1.00) | 0.98 (0.96-1.00) | 0.97 (0.95-0.98) |
|  |  |  |  |
| Notes: PRR = prevalence-rate ratios. 95% CI = 95% confidence interval All models were adjusted for women´s age and education with sub-cities educational attainment, cities GDP and population size as random effects. a. Educational attainment. Sum of z-scores of a. Proportion of the population aged 25 or older who completed secondary education or above. b. Proportion of the population aged 25 or older who completed university education or above.  b.  GDP (gross domestic product) per capita for each city per population for 2015. c. City population size for 2015 is log transformed. |  |  |  |

Supplementary Figure 1: Relation between cesarean section rates and socioeconomic variables. 

Note:

* 2015-2017 for Guatemala

Population educational attainment uses two census measured at population level: a. Proportion of the population aged 25 or older who completed secondary education or above. b. Proportion of the population aged 25 or older who completed university education or above. Measure was created with standardized z-scores of the two variables.
